# Supplementary material for: Predicting aquatic development and mortality rates of Aedes aegypti
Source: PLoS One. 2019 May 21;14(5):e0217199. doi: 10.1371/journal.pone.0217199 (PMC6528993; doi:10.1371/journal.pone.0217199)
Supplement: S1 Table — (DOCX) [file pone.0217199.s001.docx]

*Table S1. Analysis of predictor estimates for average pupation rate*

| **Coefficient** | **Estimate** | **Standard Error** | **t value** | **p value** |
| --- | --- | --- | --- | --- |
| $B_{0}$ | 1.203x10^-3^ | 2.599x10^-4^ | 4.629 | 4.43x10^-6^ |
| $B_{1}$ | 4.567x10^-4^ | 1.693x10^-5^ | 26.977 | < 2x10^-16^ |
| $B_{2}$ | 5.485x10^-4^ | 5.530x10^-5^ | 9.918 | < 2x10^-16^ |
| $B_{3}$ | 1.259x10^-1^ | 6.796x10^-3^ | 18.522 | < 2x10^-16^ |
| $B_{4}$ | 8.573x10^-3^ | 4.596x10^-4^ | 18.653 | < 2x10^-16^ |
| $B_{5}$ | -5.028x10^-3^ | 4.445x10^-4^ | -11.313 | < 2x10^-16^ |
| $B_{6}$ | -3.411x10^-3^ | 4.238x10^-4^ | -8.050 | 3.83x10^-15^ |
| $B_{12}$ | -2.133x10^-5^ | 1.909x10^-6^ | -11.176 | < 2x10^-16^ |
| $B_{13}$ | -1.027x10^-4^ | 5.064x10^-6^ | -20.271 | < 2x10^-16^ |
| $B_{23}$ | 1.035x10^-5^ | 1.889x10^-5^ | 0.548 | 0.584 |
| $B_{123}$ | -5.453x10^-7^ | 6.691x10^-7^ | -0.815 | 0.415 |
