# Supplementary material for: Predicting aquatic development and mortality rates of Aedes aegypti
Source: PLoS One. 2019 May 21;14(5):e0217199. doi: 10.1371/journal.pone.0217199 (PMC6528993; doi:10.1371/journal.pone.0217199)
Supplement: S2 Table — (DOCX) [file pone.0217199.s002.docx]

*Table S2. Analysis of predictor estimates for average adult emergence rate*

| **Coefficient** | **Estimate** | **Standard Error** | **t value** | **p value** |
| --- | --- | --- | --- | --- |
| $B_{0}$ | -1.961x10^-4^ | 1.604x10^-4^ | -1.223 | 0.222 |
| $B_{1}$ | 3.634x10^-4^ | 1.045x10^-5^ | 34.789 | < 2x10^-16^ |
| $B_{2}$ | 3.575x10^-4^ | 3.412x10^-5^ | 10.475 | < 2x10^-16^ |
| $B_{3}$ | 8.520x10^-2^ | 4.194x10^-3^ | 20.316 | < 2x10^-16^ |
| $B_{4}$ | 5.101x10^-3^ | 2.836x10^-4^ | 17.990 | < 2x10^-16^ |
| $B_{5}$ | -3.218x10^-3^ | 2.743x10^-4^ | -11.732 | < 2x10^-16^ |
| $B_{6}$ | -1.832x10^-3^ | 2.615x10^-4^ | -7.008 | 5.94x10^-12^ |
| $B_{12}$ | -1.420x10^-5^ | 1.178x10^-6^ | -12.058 | < 2x10^-16^ |
| $B_{13}$ | -6.841x10^-5^ | 3.125x10^-6^ | -21.894 | < 2x10^-16^ |
| $B_{23}$ | 8.593x10^-7^ | 1.166x10^-5^ | 0.074 | 0.941 |
| $B_{123}$ | -1.423x10^-7^ | 4.128x10^-7^ | -0.345 | 0.730 |
