# Supplementary material for: Predicting aquatic development and mortality rates of Aedes aegypti
Source: PLoS One. 2019 May 21;14(5):e0217199. doi: 10.1371/journal.pone.0217199 (PMC6528993; doi:10.1371/journal.pone.0217199)
Supplement: S3 Table — (DOCX) [file pone.0217199.s003.docx]

*Table S3. Analysis of predictor estimates for average juvenile mortality rate*

| **Coefficient** | **Estimate** | **Standard Error** | **t value** | **p value** |
| --- | --- | --- | --- | --- |
| $B_{0}$ | -3.858x10^-2^ | 4.093x10^-3^ | -9.427 | < 2x10^-16^ |
| $B_{1}$ | 1.504x10^-3^ | 2.666x10^-4^ | 5.642 | 2.49x10^-8^ |
| $B_{2}$ | 3.656x10^-3^ | 8.708x10^-4^ | 4.198 | 3.06x10^-5^ |
| $B_{3}$ | -3.599x10^-2^ | 1.070x10^-1^ | -0.336 | 0.737 |
| $B_{4}$ | 3.493x10^-2^ | 7.237x10^-3^ | 4.827 | 1.72x10^-6^ |
| $B_{5}$ | 2.438x10^-3^ | 6.999x10^-3^ | 0.348 | 0.728 |
| $B_{6}$ | 4.281x10^-2^ | 6.673x10^-3^ | 6.415 | 2.67x10^-10^ |
| $B_{12}$ | -1.341x10^-4^ | 3.005x10^-5^ | -4.460 | 9.62x10^-6^ |
| $B_{13}$ | 1.185x10^-4^ | 7.974x10^-5^ | 1.486 | 0.138 |
| $B_{23}$ | -1.327x10^-3^ | 2.975x10^-4^ | -4.460 | 9.63x10^-6^ |
| $B_{123}$ | 6.029x10^-5^ | 1.054x10^-5^ | 5.722 | 1.59x10^-8^ |
