# Supplementary material for: Predicting aquatic development and mortality rates of Aedes aegypti
Source: PLoS One. 2019 May 21;14(5):e0217199. doi: 10.1371/journal.pone.0217199 (PMC6528993; doi:10.1371/journal.pone.0217199)
Supplement: S4 Table — (DOCX) [file pone.0217199.s004.docx]

*Table S4: Paired t-test and confidence interval for average pupation rate*

| Statistic | N | Mean | St. Deviation | SE Mean |
| --- | --- | --- | --- | --- |
| Experimental | 225 | 0.006729 | 0.001482 | 0.00099 |
| Predicted | 225 | 0.006716 | 0.001703 | 0.000114 |
| Difference | 225 | 0.000013 | 0.00576 | 0.00039 |

95% CI for mean difference: (-6.3x10^-5^, 8.9x10^-5^)

t-test of mean difference: t-value = 0.34 p-value = 0.731
