# Supplementary material for: Predicting aquatic development and mortality rates of Aedes aegypti
Source: PLoS One. 2019 May 21;14(5):e0217199. doi: 10.1371/journal.pone.0217199 (PMC6528993; doi:10.1371/journal.pone.0217199)
Supplement: S5 Table — (DOCX) [file pone.0217199.s005.docx]

*Table S5: Paired t-test and confidence interval for average adult emergence rate*

| Statistic | N | Mean | St. Deviation | SE Mean |
| --- | --- | --- | --- | --- |
| Experimental | 225 | 0.005215 | 0.000962 | 0.000064 |
| Predicted | 225 | 0.005215 | 0.001162 | 0.000078 |
| Difference | 225 | 0.00000 | 0.000406 | 0.000027 |

95% CI for mean difference: (-5.3x10^-5^, 5.4x10^-5^)

t-test of mean difference: t-value = 0.01 p-value = 0.995
