# Supplementary material for: Predicting aquatic development and mortality rates of Aedes aegypti
Source: PLoS One. 2019 May 21;14(5):e0217199. doi: 10.1371/journal.pone.0217199 (PMC6528993; doi:10.1371/journal.pone.0217199)
Supplement: S6 Table — (DOCX) [file pone.0217199.s006.docx]

*Table S6: Paired t-test and confidence interval for average juvenile mortality rate*

| Statistic | N | Mean | St. Deviation | SE Mean |
| --- | --- | --- | --- | --- |
| Experimental | 225 | 0.02143 | 0.01435 | 0.00096 |
| Predicted | 225 | 0.02182 | 0.01757 | 0.00117 |
| Difference | 225 | -0.000395 | 0.009516 | 0.000636 |

95% CI for mean difference: (-1.648x10^-3^, 8.57 x10^-4^)

t-test of mean difference: t-value = -0.62 p-value = 0.535
